# Coronavirus disease 2019 (COVID-19) and spatial control in times of pandemic

**DOI:** 10.1017/ice.2020.1424

**Published:** 2021-01-11

**Authors:** Marcelo Carneiro, Camilo Darsie, Janine Koepp, Andreia Rosane de Moura Valim, Lia Gonçalves Possuelo, Marina Weiss Kist, Eliane Carlosso Krummenauer, Rochele Mosman de Menezes, Lea Vargas, Pola A Brenner

**Affiliations:** 1 Hospital Infection and Epidemiology Control Committee, Hospital Santa Cruz, Rio Grande do Sul, Brazil; 2 School of Medicine, Universidade de Santa Cruz do Sul, Rio Grande do Sul, Brazil; 3 School of Nursing, Universidade de Santa Cruz do Sul, Rio Grande do Sul, Brazil; 4 *Strictu Sensu* Program in Health Promotion, Universidade de Santa Cruz do Sul, Rio Grande do Sul, Brazil; 5 *Strictu Sensu* Program in Education, Universidade de Santa Cruz do Sul, Rio Grande do Sul, Brazil; 6 Consórcio Intermunicipal de Saúde (CISVALE), Santa Cruz do Sul, Rio Grande do Sul, Brazil; 7 *Strictu Sensu* Program in Healthcare-Associated Infections, Universidad de Valparaiso, Valparaíso, Chile

*To the Editor*—Coronavirus-19 infection (COVID-19) occurs through the spread of the severe acute respiratory syndrome coronavirus-2 (SARS-CoV-2) among individuals, mainly by direct contact or droplet transmission when infected individuals cough or sneeze. Pulmonary epithelial cells are the main target of the virus.^1^ The worldwide proliferation of this virus has caused a pandemic capable of changing paradigms related to healthcare delivery, and the resources needed to cope with the disease have directly influenced the safety of medical care offered to individuals on a global scale. The purpose of interventions, such as social distancing, is to guarantee broad and safe assistance to the global population and to minimize uncontrolled viral spread. Notably, globalization and, consequently, the great movement of people, animals, and products across geophysical and political boundaries that has characterized and facilitated modern life, has also increased the spread of diseases, facilitating the second viral pandemic in this century.^2,3^

Unlike the 2009 influenza pandemic, the emphasis on spatial control with the COVID-19 pandemic has interfered with social, political, and economic relationships. This disruption has resulted in the destabilization of global geopolitics and the economy. The important concepts of space management and educational actions related to disease control originally emerged from previous health crises. These interventions can be considered geobiopolitical strategies, that is, actions directed at the control of life through geopolitical demands.^4^ At first, science was able to control contagious diseases and increase the survival of the populations exposed to them through biology (eg, isolation of populations by natural geographical barriers). However, with the increase in a mobile and diverse global population with different lifestyles and the inequalities related to health care, the dissemination of new infectious agents has occurred, primarily through the transmission of disease-producing viruses that have escaped the usual biological control mechanisms.

As more people worldwide aspire to better lives, it is no longer sufficient to control infections at any cost. We must learn how our interventions to control diseases not only impact population but also the lives of individuals. Such strategies are characterized as biopolitical actions associated with biopower. Biopower can be understood as the inclusion of biology in the context of politics. Using biopower, governments start to calculate and act on health issues aiming to strengthen the lives of populations as a group of individuals. Over the years, strategies to save and maintain the quality of human life have been highlighted. Biopower comprises the relationships among 3 dimensions: (1) universally held truths regarding the value of the individual and their quality of life and authorities willing to defend those truths; (2) different strategies that allow interventions in favor of life or death to occur; and finally, (3) allowing individuals to subjectively choose and act on their own behalf incorporating these universal truths.

In the case of COVID-19, the subjectivity regarding the importance of social isolation stands out, being considered a “norm” of safety to prevent infection or disease. Although a rational approach, considering the lack of actually efficient and/or sufficient treatment structures, this strategy generates antagonistic feelings depending on the experiences of each person. This strategy may engender fear, mistrust, solidarity, and/or empathy in different scenarios and among different people.^5^ The disclosure of statistical data about the efficacy of social isolation practices does not guarantee that the real benefits or harms of epidemiological surveillance will be understood. The structure of these truths is a fragile one, and the responses of different cultures may not be predictable or standardized. Indexes related to the efficiency of the social isolation strategy tend to reinforce the idea of isolation as the most appropriate alternative, thus imposing the truth on the populations constrained.^6^ Thus, when thought of as a global recommendation that is confirmed as “numerically appropriate,” the social isolation discourse subjectifies the individuals who, for different reasons, are pressured to comply with the norm.

There are reasons to make social isolation more flexible. Reasons supported by other statistical data that highlight possible problems caused by this practice (eg, anxiety, economic downturn, and domestic violence) might lead to a different strategy, even if it is not “the best choice.” Individuals live and are inseparable from their environments, therefore, experience different spatialities. Thus, the places they live produce different ways of being.^4^ Thus, far from an attempt to question the validity of social isolation, the importance of questioning the effects of such a biopolitical strategy emerges. Whether a biopolitical strategy that recommends social isolation, as it is occurring, will be successful in preventing the spread of the disease remains unknown, and the real impact, in all spheres of life of different individuals, remains uncertain. Isolation of populations may successfully prevent the spread of infection but may also result in tensions and the deterioration of the public mental health.
